# Serum Bile Acid Concentrations in Dogs with Exocrine Pancreatic Insufficiency

**DOI:** 10.3390/ani16182941

**Published:** 2026-09-18

**Authors:** Joerg M. Steiner, Lydie Humbert, Dominique Rainteau, Amanda B. Blake, Frédéric Carrière

**Affiliations:** 1 Gastrointestinal Laboratory, Department of Small Animal Clinical Sciences, College of Veterinary Medicine and Biomedical Sciences, Texas A&M University, College Station, TX 77843-4474, USA; ablake@cvm.tamu.edu; 2 Clinical Metabolomics Department, Research Center Saint-Antoine, Saint-Antoine Hospital, Sorbonne University, Inserm, 75012 Paris, France; lydie.humbert@gmail.com (L.H.); dr.rainteau@gmail.com (D.R.); 3 Bioenergetics and Protein Engineering Laboratory, French National Center for Scientific Research, Aix-Marseille Université, 13009 Marseille, France; carriere@imm.cnrs.fr

**Keywords:** bile acids, bile acid dysmetabolism, dysbiosis, exocrine pancreatic insufficiency, gastrointestinal, German shepherd dog

## Abstract

Exocrine pancreatic insufficiency (EPI) is a chronic gastrointestinal disease in dogs that is caused by the insufficient synthesis and secretion of pancreatic digestive enzymes by the exocrine pancreas. Previous studies have described intestinal dysbiosis in dogs with EPI and dysbiosis is often associated with bile acid dysmetabolism. This study aimed to measure the concentration of a variety of bile acids in the serum of dogs with EPI. Serum was collected from 50 dogs with a serum canine trypsin-like immunoreactivity (cTLI) concentration of less than 1 µg/L, strongly suggesting EPI, and 50 non-EPI control dogs with a serum cTLI concentration within the reference interval. Half of the dogs with EPI and half the non-EPI control dogs were German shepherd dogs and half were dogs of other breeds. There were significant differences in serum bile acid concentrations between non-EPI control dogs and dogs with EPI. Dogs with EPI had significantly higher serum concentrations of both primary and total bile acids. This study shows that EPI in dogs is associated with bile acid dysmetabolism.

## Simple Summary

Exocrine pancreatic insufficiency (EPI) is a chronic gastrointestinal disease in dogs that is caused by the insufficient synthesis and secretion of pancreatic digestive enzymes by the exocrine pancreas. Previous studies have described an abnormality of intestinal microorganisms in dogs with EPI and these abnormalities are often associated with abnormalities in bile acid metabolism. This study aimed to measure the concentration of a variety of bile acids in the serum of dogs with EPI. Serum was collected from 50 dogs suspected of having EPI and 50 dogs that were not suspected of having EPI. Half of the dogs with EPI and half the control dogs were German shepherd dogs and half were dogs of other breeds. There were significant differences in serum bile acid concentrations between non-EPI control dogs and dogs with EPI. Dogs with EPI had significantly higher serum concentrations of primary and total bile acid concentrations. This study shows that EPI in dogs is associated with bile acid dysmetabolism.

## 1. Introduction

Exocrine pancreatic insufficiency (EPI) is a chronic gastrointestinal condition that is due to insufficient synthesis and secretion of digestive enzymes by the exocrine pancreas [1]. EPI in German shepherd dogs (GSDs) is most frequently caused by pancreatic acinar atrophy (PAA), a condition where dogs are born with a normal pancreas, but the pancreas gradually atrophies in early adulthood, leading to clinical signs at around 2–3 years of age [1,2,3]. In sharp contrast, EPI in other breeds of dogs is caused by chronic pancreatitis and can develop at any age [1]. The two conditions could theoretically be distinguished based on histopathology, where dogs with PAA show no inflammation and fibrosis of the acinar tissue, while dogs with chronic pancreatitis show inflammatory infiltration of the pancreas and various degrees of fibrosis [2]. Both PAA and chronic pancreatitis, once they have led to EPI, are associated with the same clinical signs, such as weight loss, soft voluminous stools, borborygmus, flatulence, and a poor hair coat [1]. Many dogs with EPI, regardless of the underlying cause of the disease, respond to pancreatic enzyme replacement therapy (PERT), but both conditions might be associated with different pathophysiological changes in the gut [4].

Several years ago, it was reported that German shepherd dogs with EPI have small intestinal bacterial overgrowth (SIBO), but it has since been clarified that this secondary disturbance of the intestinal microbiome is better referred to as intestinal dysbiosis [5]. It has also been demonstrated that, using untargeted metabolomics, EPI is associated with a wide variety of metabolic changes [6]. Furthermore, intestinal dysbiosis is associated with bile acid dysmetabolism as the metabolism of bile acids in the intestinal lumen mainly relies on bacterial metabolism [7]. Thus, it would be expected that dogs with EPI show significant intestinal and systemic bile acid dysmetabolism.

**Figure 1:**
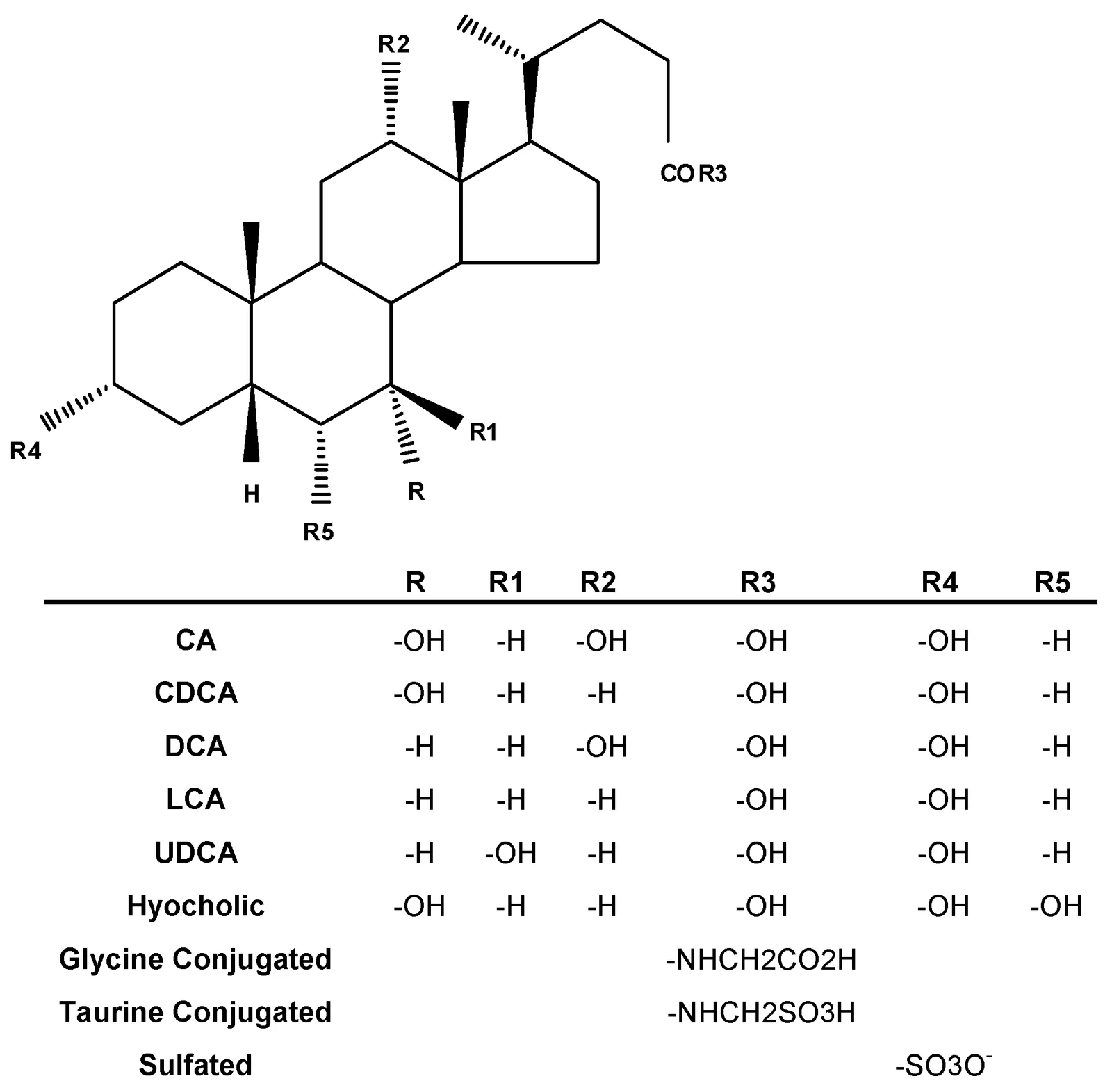
Bile Acids: This figure shows the biochemical structure of some of the wide variety of bile acids that were measured along with their glycine- and taurine-conjugated counterparts. Sulfated BA included unconjugated CDCA-3S, DCA-3S, and CA-3S, along with unconjugated and conjugated UDCA-3S and LCA-3S. The core of the molecule of all bile acids is derived from cholesterol and has 5 attached groups that determine the type of BA or whether the BA is conjugated or unconjugated (Figure reproduced with permission: Humbert et al. J. Chromatogr. B, 2012) [8].

Therefore, the goal of this study was to measure and compare serum concentrations of a wide variety of bile acids (Figure 1) between dogs with EPI and non-EPI control dogs. Furthermore, in order to be able to demonstrate differences in the pathophysiology between PAA and chronic-pancreatitis-associated EPI, serum concentrations of bile acids were compared between dogs with a high suspicion of PAA (i.e., GSDs with a cTLI ≤ 1.0 µg/L) and those with a high suspicion of chronic pancreatitis (i.e., non-GSDs with a cTLI ≤ 1.0 µg/L) and non-EPI controls of either breed group.

## 2. Materials and Methods

### 2.1. Samples

Leftover serum samples from dogs with a high clinical suspicion of EPI were collected at the Gastrointestinal Laboratory (GI-Lab) at Texas A&M University (College Station, TX, USA) based on a serum cTLI concentration of ≤1 µg/L (reference interval at the time of this study: 5.7–45.2 µg/L; suggested cut-off value for EPI: 2.5 µg/L). Leftover serum samples were defined as those that had been sent in by veterinarians unaware of this study for the measurement of serum cTLI where at least 1 mL was left over after all requested diagnostic tests had been performed.

Serum samples from non-EPI control dogs were also collected from leftover serum samples at the GI-Lab of Texas A&M University and were identified based on having a serum cTLI concentration > 10 µg/L and serum cobalamin and folate concentrations within their respective reference intervals (RI at the time of this study for serum cobalamin: 251–908 ng/L; for serum folate: 7.7–24.4 µg/L). Importantly, all of these samples had been submitted to the GI-Lab of Texas A&M University for the purpose of diagnostic testing. Thus, it is likely that none of the dogs from whom these samples had been collected were healthy. Instead, these dogs likely showed clinical signs of chronic gastrointestinal disease. Thus, this group of dogs should not be considered as healthy controls, but as a non-EPI control group.

None of the veterinarians submitting the samples included in this study were aware of this study and thus no blood samples were collected for the purpose of this study. Also, all samples were selected by the laboratory staff unaware of the purpose of this study and all 5 investigators were blinded to the dogs whose serum was being used. Thus, no demographic or clinical data are available for the dogs whose serum sample had been included in this study.

### 2.2. Analytical Methods

A variety of serum bile acid (BA) concentrations (Figure 1) were identified and measured by LC-MS/MS at Sorbonne University, Saint Antoine, Paris, France as reported previously [9].

#### 2.2.1. Standard Solutions

Briefly, concentrated BA calibration solutions were prepared in methanol (1 mg/mL) and stored in a sealed container at −20 °C. These stock solutions were diluted to obtain calibration solutions ranging from 31.3 ng/mL to 31.3 µg/mL. Cholic acid (CA), deoxycholic acid (DCA), chenodeoxycholic acid (CDCA), ursodeoxycholic acid (UDCA), lithocholic acid (LCA), hyocholic acid (HCA), and the corresponding glycine and taurine conjugates were obtained from Sigma-Aldrich (Saint Quentin Fallavier, France). The 3-sulfate derivatives of the BAs were a generous gift from Dr. J. Goto (Niigita University of Pharmacy and Applied Life Science, Niigata, Japan). The 23-nor-5β-cholanoic acid-3α,12α diol, muricholic acid derivatives, and glycine and taurine derivatives were purchased from Steraloids Inc. (Newport, RI, USA). An internal standard solution (2 µL of 23-nor-5β-cholanoic acid-3α,12β diol at 1 mg/mL) was added to all serum samples (500–1000 µL).

#### 2.2.2. Sample Preparation

Proteins were precipitated by addition of 0.4 M ammonium carbonate for 30 min at 60 °C. The clean-up procedure was achieved by centrifugation at 4000× *g* for 10 min, followed by a solid-phase extraction. Reverse-phase Chromabond C18 cartridges (100 mg; Macherey-Nagel, Düren, Germany) were prewashed with 5 mL of methanol and 5 mL of water was loaded onto the cartridge before the sample. The subsequent steps were processed on a vacuum manifold designed for solid-phase extraction. The cartridge was rinsed with 20 mL of water, followed by 10 mL of hexane to discard neutral lipids, followed again by 20 mL of water. BAs were eluted in methanol, eluates were dried under a nitrogen stream at 50 °C, and the residues were dissolved in 150 µL of methanol.

#### 2.2.3. HPLC-MS/MS Analysis

Five microliters of the extracted BA samples were injected into the HPLC-MS/MS system. The chromatographic separation of BAs was carried out on a reverse-phase column (Restek C18 Pinnacle II, 250 × 3.2 mm, 5 µmol; Restek, Lisses, France) at 35 °C on a HPLC system (Agilent 1100 HPLC; Agilent, Massy, France). The column was initially equilibrated with a 65/35 (*v*/*v*) mixture of 15 mM of aqueous ammonium acetate, pH 5.3 and methanol. Elution of BAs was achieved by increasing the proportion of methanol from 35/65 to 95/5 (*v*/*v*). Simultaneously, the flow rate was increased from 0.3 to 0.5 mL/min over a period of 30 min. The HPLC column eluates were infused into the ESI source of a triple quadrupole mass spectrometer (QTRAP 2000; Applied Biosystems-SCIEX, Concord, ON, Canada). Electrospray ionization was set in the negative mode. Nebulizer, curtain, and heater nitrogen gases were set at 40, 20, and 40 (arbitrary units), respectively. The temperature for the evaporation gas (nitrogen) was set at 400 °C. The ion spray, declustering, and entrance potentials were set at −4500 V, −60 V, and −10 V, respectively. The MS/MS detection was operated with a unit resolution in the multiple reaction monitoring mode. The dwell time for each transition was set at 70 ms. Multiple reaction monitoring was performed by an examination of the transition reactions from precursor-conjugated BA to product fragment ions after collision-induced dissociation: the sulfite (*m*/*z* 80, SO_3_ fragment anion cleaved from taurine) and glycine moieties (*m*/*z* 74), respectively, for taurine-conjugated and glycine-conjugated BAs. Sulfo-conjugates were identified by the sulfuric anion (*m*/*z* 97, HSO_4_). No specific fragment ions were observed for unconjugated BAs, except those corresponding to loss of water, but those fragments were not reliable for quantification. Therefore, we used selected ion-monitoring mode for quantifying unconjugated BAs, with mono-, di-, and tri-hydroxylated BAs scanned at *m*/*z* 375, 391, and 407, respectively. The 23-nor-5β-cholanoic acid-3α,12αδιολ (*m*/*z* 377) was used as the internal standard for normalization. The method for BA analysis was validated according to Humbert et al., who previously have shown that similar values are obtained with one of three different internal standards (23-nor-5β-cholanoic acid-3α,12α diol, ursodeoxycholic-2,2,4,4-d4 acid, and lithocholic-2,2,4,4-d4 acid) with distinct hydrophobicity and retention times [8].

### 2.3. Calculation of Bile Acid Group Results

Total bile acids were calculated from the sum of all BAs measured. Primary BAs were calculated as the sum of all BAs that were measured that are hydroxylated at the C7 position in the liver, whereas secondary BAs were calculated as the sum of all BAs that were measured that did not have a hydroxyl group at the C7 position, resulting from 7-dehydroxylation by the intestinal microbiota.

### 2.4. Statistical Analyses

For the initial statistical analyses, 2 separate datasets were formed: 49 non-EPI control dogs and 50 dogs with EPI, regardless of breed, and both datasets were evaluated for normality using the D’Agostino & Pearson test. Serum bile acid concentrations were compared between the two groups using the Mann–Whitney test if at least one of the two data sets failed normality and the two-sided *t*-test if both datasets passed normality testing. Then the two datasets were split up based on breed and 4 separate datasets were formed: EPI-GSD, non-EPI-GSD, EPI-non-GSD, and non-EPI-non-GSD. Once again, all datasets were evaluated for normality using the D’Agostino & Pearson test. Serum bile acid concentrations were compared between the 4 groups using the Kruskal–Wallis test if any one of the datasets failed normality testing and the one-way ANOVA if all datasets passed normality testing. All statistical analyses were performed using GraphPad Prism 9.0 (San Diego, CA, USA) and significance was set at *p* < 0.05.

## 3. Results

Serum samples were available from a total of 99 dogs. One serum sample from one of the German shepherd dogs without EPI that had been identified was not available for analysis. Therefore, we analyzed 25 serum samples from German shepherd dogs with EPI (EPI-GSD), 24 German shepherd dogs without EPI (non-EPI-GSD), 25 dogs of non-German-shepherd dog breed dogs with EPI (EPI-non-GSD), and 25 dogs of non-German-shepherd dog breeds without EPI (non-EPI-non-GSD).

When analyzing two groups, all data sets failed normality testing and therefore Mann–Whitney tests were used for these comparisons. Additionally, when comparing four groups, all data sets failed normality testing and therefore Kruskal–Wallis tests were used for these comparisons.

### 3.1. Total Bile Acids

**Figure 2:**
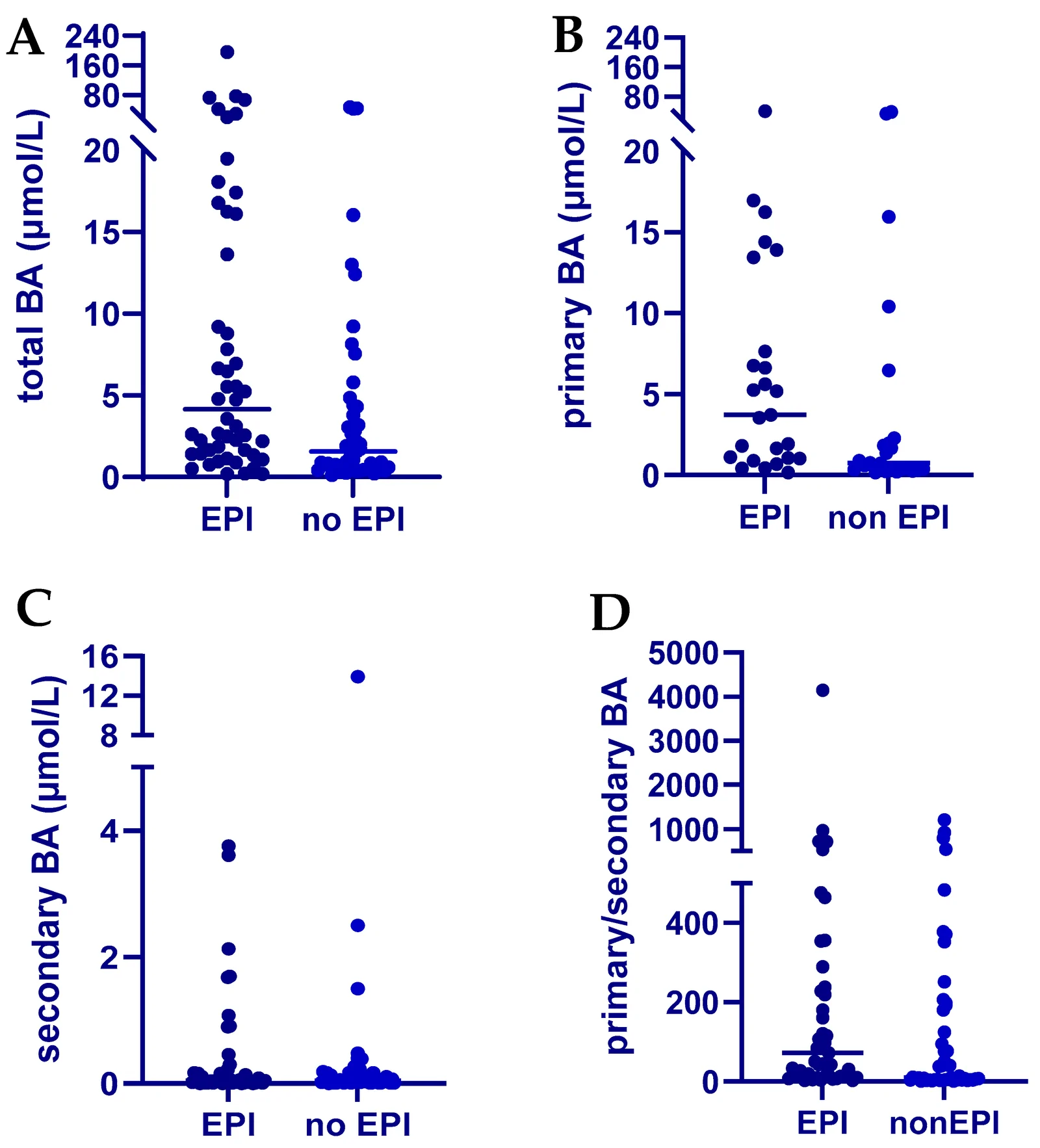
Serum concentrations of total bile acids, primary bile acids, secondary bile acids, and primary to secondary bile acid ratios: This figure shows serum concentrations of total bile acids (**A**), primary bile acids (**B**), secondary bile acids (**C**), and primary to secondary bile acid ratios (**D**) in 50 dogs with EPI and 49 dogs without EPI of various breeds. Total serum bile acid concentrations, primary bile acid concentrations, and primary to secondary bile acid ratios were significantly higher in dogs with EPI than in those without. No statistically significant difference in serum secondary bile acid concentrations could be observed between the two groups.

Serum total bile acid concentrations were significantly different between the two groups of dogs with dogs with EPI having a median serum total bile acid concentration of 4.1 µmol/L (range: 0.2 to 196.0 µmol/L) and dogs without EPI having a median serum total bile acid concentration of 1.6 µmol/L (range: 0.1 to 47.7 µmol/L; *p*-value: 0.0022; Table 1; Figure 2).

**Figure 3:**
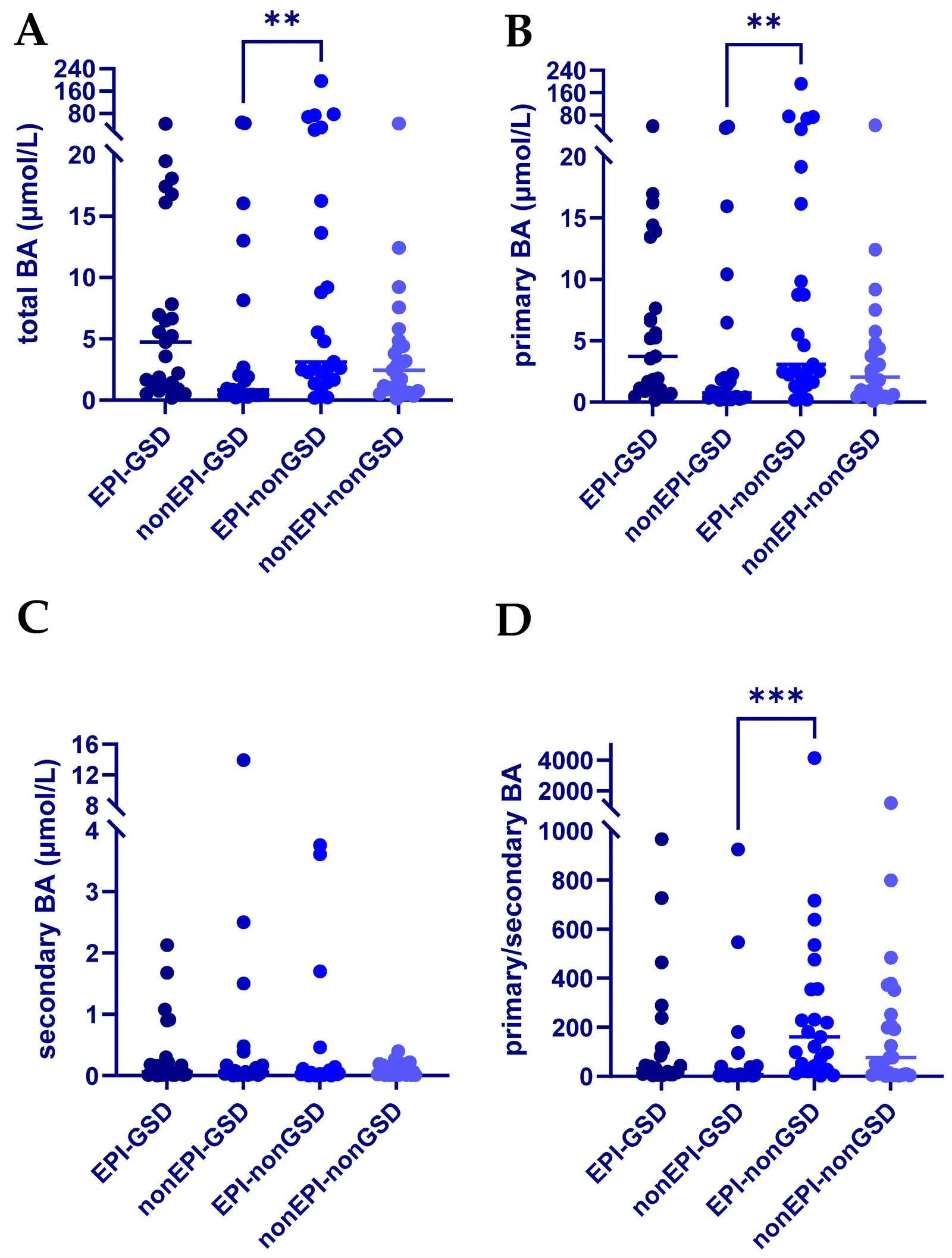
Serum concentrations of total bile acids, primary bile acids, secondary bile acids, and primary to secondary bile acid ratios: This figure shows serum concentrations of total bile acids (**A**), primary bile acids (**B**), secondary bile acids (**C**), and primary to secondary bile acid ratios (**D**) in 25 GSD with EPI, 24 GSD without EPI, 25 dogs of other breeds with EPI, and 25 dogs of other breeds without EPI. Total serum bile acid concentrations, primary bile acid concentrations, and primary to secondary bile acid ratios were significantly different between the four groups, but no statistically significant difference in serum secondary bile acid concentrations could be observed between the four groups. Significant differences between groups were determined by a Kruskal–Wallis test followed by Dunn’s post hoc tests. Data points represent individual values; horizontal bars indicate group medians. * *p* < 0.05, ** *p* < 0.01, *** *p* < 0.001.

Serum total bile acid concentrations were statistically significantly different between the four groups of dogs (*p*-value: 0.0097; Figure 3). Median (range) serum total bile acid concentrations were 4.7 µmol/L (0.2–42.1 µmol/L) for EPI-GSD, 0.8 µmol/L (0.2–47.7 µmol/L) for non-EPI-GSD, 3.1 µmol/L (0.2–196.0 µmol/L for EPI-non-GSD, and 2.5 µmol/L (0.1–43.6 µmol/L) for non-EPI-non-GSD. Dunn’s multiple comparison tests only identified a statistically significant difference in serum total bile acid concentrations between non-EPI-GSD and EPI-non-GSD (*p*-value: 0.0096).

### 3.2. Primary Bile Acid Profiles

Serum total primary bile acid concentrations represent the sum of all bile acids measured with a C7 hydroxyl group, and they were significantly different between the two groups of dogs with dogs with EPI having a median serum primary bile acid concentration of 3.7 µmol/L (range: 0.2 to 40.5 µmol/L) and dogs without EPI having a median serum primary bile acid concentration of 0.7 µmol/L (range: 0.2 to 38.4 µmol/L; *p*-value: 0.0161; Table 1, Figure 2). Serum total primary bile acid concentrations were statistically significantly different between the four groups of dogs (*p*-value: 0.0072; Figure 3). Median (range) serum concentrations were 3.7 µmol/L (0.2–40.5 µmol/L) for EPI-GSD, 0.7 µmol/L (0.2–38.4 µmol/L) for non-EPI-GSD, 3.1 µmol/L (0.2–192.1 µmol/L) for EPI-non-GSD, and 2.0 µmol/L (0.1–43.3 µmol/L) for non-EPI-non-GSD. Dunn’s multiple comparison tests only identified a statistically significant difference in serum total primary bile acid concentrations between non-EPI-GSD and EPI-non-GSD (*p*-value: 0.0069).

**Figure 4:**
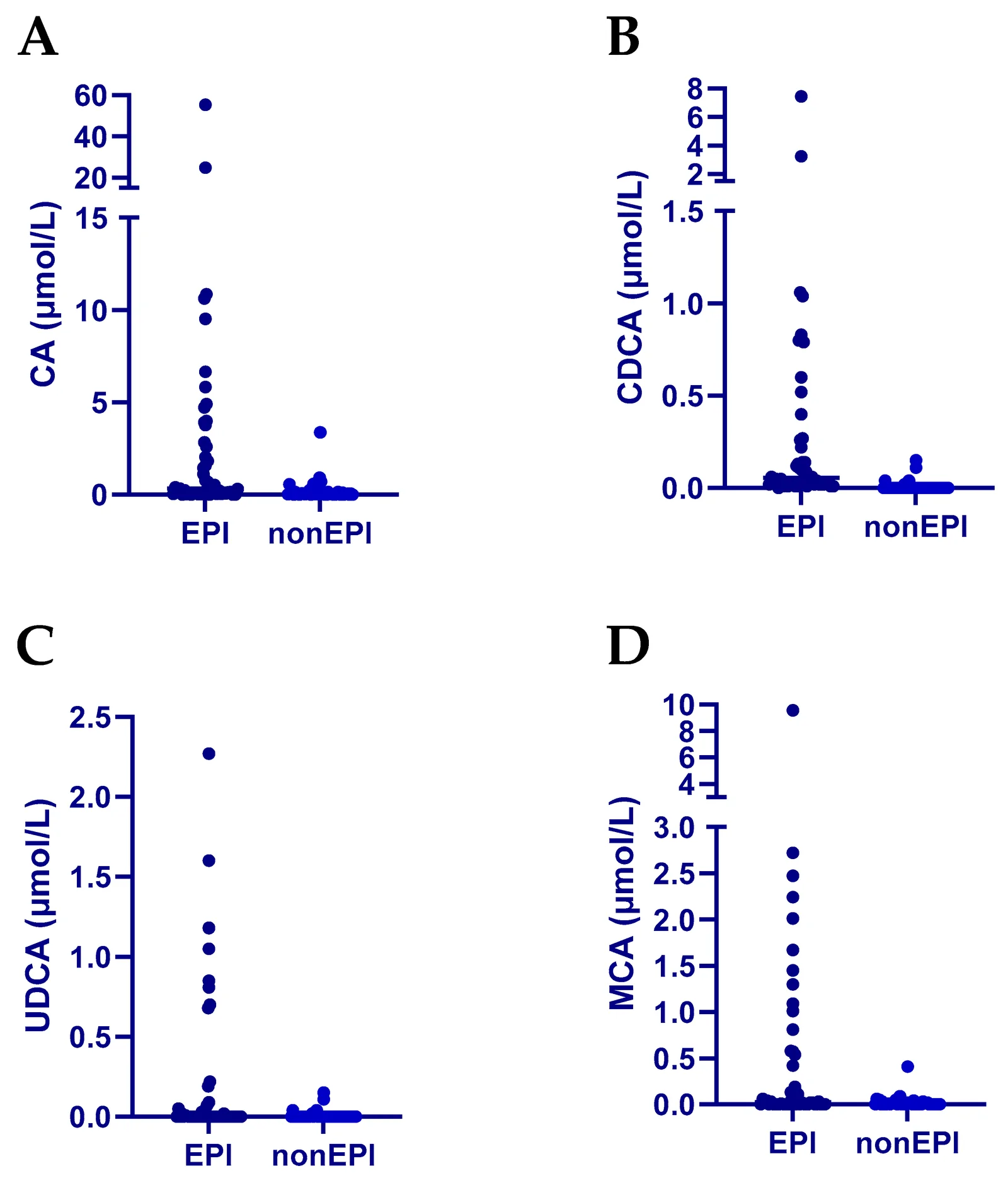
Serum concentrations of primary bile acids cholic acid, chenodeoxycholic acid, ursodeoxycholic acid, and muricholic acid: This figure shows serum concentrations of cholic acid (**A**), chenodeoxycholic acid (**B**), ursodeoxycholic acid (**C**), and muricholic acid (**D**) in 50 dogs with EPI and 49 dogs without EPI of various breeds. Cholic acid, chenodeoxycholic acid, ursodeoxycholic acid, and muricholic acid were all significantly higher in dogs with EPI than in those without.

Serum cholic acid (CA) concentrations were significantly different between the two groups of dogs, with dogs with EPI having a median of 0.33 µmol/L (range: 0.00 to 55.34 µmol/L) and dogs without EPI having a median of 0.06 µmol/L (range: 0.00 to 3.38 µmol/L; *p*-value: <0.0001; Table 1, Figure 4).

Serum CA concentrations were statistically significantly different between the four groups of dogs (*p*-value: <0.0001). Dunn’s multiple comparison analyses showed a significant difference between EPI-GSD and non-EPI-GSD or non-EPI-non-GSD and between EPI-non-GSD and non-EPI-non-GSD.

While all serum samples from dogs with EPI had measurable chenodeoxycholic acid (CDCA) concentrations, most serum samples from those in the non-EPI group had concentrations that were below the lower limit of detection (43/49). Serum CDCA concentrations were significantly different between the two groups of dogs, with dogs with EPI having a median of 0.06 µmol/L (range: 0.00 to 7.44 µmol/L) and dogs without EPI having a median of 0.00 µmol/L (range: 0.00 to 0.15 µmol/L; *p*-value: <0.0001; Figure 4).

Serum CDCA concentrations were statistically significantly different between the four groups of dogs (*p*-value: 0.05). Dunn’s multiple comparison analyses showed a significant difference between EPI-GSD and non-EPI-GSD or non-EPI-non-GSD and between EPI-non-GSD or non-EPI-non-GSD and non-EPI-GSD.

Many serum samples from either dogs with EPI (31/50) or those without EPI (43/49) had serum ursodeoxycholic acid (UDCA) concentrations that were below the lower limit of detection. Serum UDCA concentrations were significantly different between the two groups of dogs, with dogs in both groups having a median of 0.00 µmol/L (ranges: 0.00 to 2.27 µmol/L for dogs with EPI and 0.00 to 0.15 µmol/L for those without EPI; *p*-value: 0.0023; Figure 4).

Serum UDCA concentrations were statistically significantly different between the four groups of dogs (*p*-value: 0.0003). Dunn’s multiple comparison analyses showed a significant difference between EPI-GSD and non-EPI-GSD, EPI-non-GSD, and non-EPI-non-GSD.

Serum MCA concentrations were significantly different between the two groups of dogs, with dogs with EPI having a median of 0.03 µmol/L (range: 0.00 to 9.55 µmol/L) and dogs without EPI having a median of 0.00 µmol/L (range: 0.00 to 0.41 µmol/L; *p*-value: 0.0018; Table 1, Figure 4).

Serum MCA concentrations were statistically significantly different between the four groups of dogs (*p*-value: 0.0083). Dunn’s multiple comparison analyses showed a significant difference between EPI-GSD and non-EPI-GSD or non-EPI-non-GSD.

### 3.3. Secondary Bile Acid Profiles

Serum total secondary bile acid concentrations, represented by the sum of all BA without a C7 hydroxyl group, were not significantly different between all dogs with EPI and all dogs without EPI (Table 1, Figure 2). When comparing the four groups separately, there was no statistically significant difference (Figure 3).

Serum deoxycholic acid (DCA) concentrations were not significantly different between the two groups of dogs, with a median of 0.02 µmol/L (range: 0.00 to 2.35 µmol/L) for EPI dogs and 0.01 µmol/L (range: 0.00 to 1.58 µmol/L) for non-EPI dogs. Serum DCA concentrations were not statistically significantly different between the four groups of dogs.

### 3.4. Primary to Secondary Bile Acid Ratios

Serum primary to secondary bile acid ratios were significantly different between the two groups of dogs with dogs with EPI having a median ratio of 73 (range: 3 to 4148) and dogs without EPI having a median ratio of 11 (range: 2 to 1213; *p*-value: 0.0152; Table 1, Figure 2). Serum primary to secondary bile acid ratios were statistically significantly different between the four groups of dogs (*p*-value: 0.0019; Figure 3). Median (range) serum primary to secondary bile acid ratios were 32 (4–967) for EPI-GSD, 8 (2–925) for non-EPI-GSD, 160 (3–4148) for EPI-non-GSD, and 76 (1–1213) for non-EPI-non-GSD. Dunn’s multiple comparison tests only identified a statistically significant difference in serum primary to secondary bile acid ratios between non-EPI-GSD and EPI-non-GSD (*p*-value: 0.0008).

### 3.5. Glycine-Conjugated Bile Acids

Many serum samples from either dogs with EPI (24/50) or those non-EPI (16/49) had serum glycoursodeoxycholic acid (GUDCA) concentrations that were below the lower limit of detection. Serum GUDCA concentrations were not significantly different between the two groups of dogs, with a median of 0.00 µmol/L for both groups (ranges: 0.00 to 0.02 µmol/L for dogs with EPI and 0.00 to 0.03 µmol/L for non-EPI dogs). Data for the subgroups were not further analyzed due to the low concentrations observed.

Most serum samples from dogs with EPI (43/50) and many of those without EPI (34/49) had serum glycochenodeoxycholic acid (GCDCA) concentrations that were below the lower limit of detection. Serum GCDCA concentrations were not significantly different between the two groups of dogs, with a median of 0.00 µmol/L for both groups (ranges: 0.00 to 0.03 µmol/L for dogs with EPI and 0.00 to 0.06 µmol/L for non-EPI dogs). Data for the subgroups were not further analyzed due to the low concentrations observed.

Many serum samples from either dogs with EPI (39/50) or those without EPI (39/49) had serum glycocholic acid (GCA) concentrations that were below the lower limit of detection. Serum GCA concentrations were not significantly different between the two groups of dogs, with a median of 0.00 µmol/L for both groups (ranges: 0.00 to 0.03 µmol/L for dogs with EPI and 0.00 to 0.11 µmol/L for non-EPI dogs). Data for the subgroups were not further analyzed due to the low concentrations that had been observed.

### 3.6. Taurine-Conjugated Bile Acids

Serum total taurine-conjugated bile acid concentrations were significantly different between the two groups of dogs, with dogs with EPI having a median concentration of 2.5 µmol/L (range: 0.1 to 182.2 µmol/L) and dogs without EPI having a median concentration of 1.1 µmol/L (range: 0.1 to 45.9 µmol/L; *p*-value: 0.0205; Table 1).

Serum total taurine-conjugated bile acid concentrations were statistically significantly different between the four groups of dogs (*p*-value: 0.0151). Median (range) concentrations were 1.5 µmol/L (0.2–12.6 µmol/L) for EPI-GSD, 0.7 µmol/L (0.2–45.9 µmol/L) for non-EPI-GSD, 3.0 µmol/L (0.1–182.2 µmol/L) for EPI-non-GSD, and 2.1 (0.1–43.3 µmol/L) for non-EPI-non-GSD. Dunn’s multiple comparison tests identified a statistically significant difference in serum taurine-conjugated bile acid concentrations between non-EPI-GSD and EPI-non-GSD (*p*-value: 0.0074).

**Figure 5:**
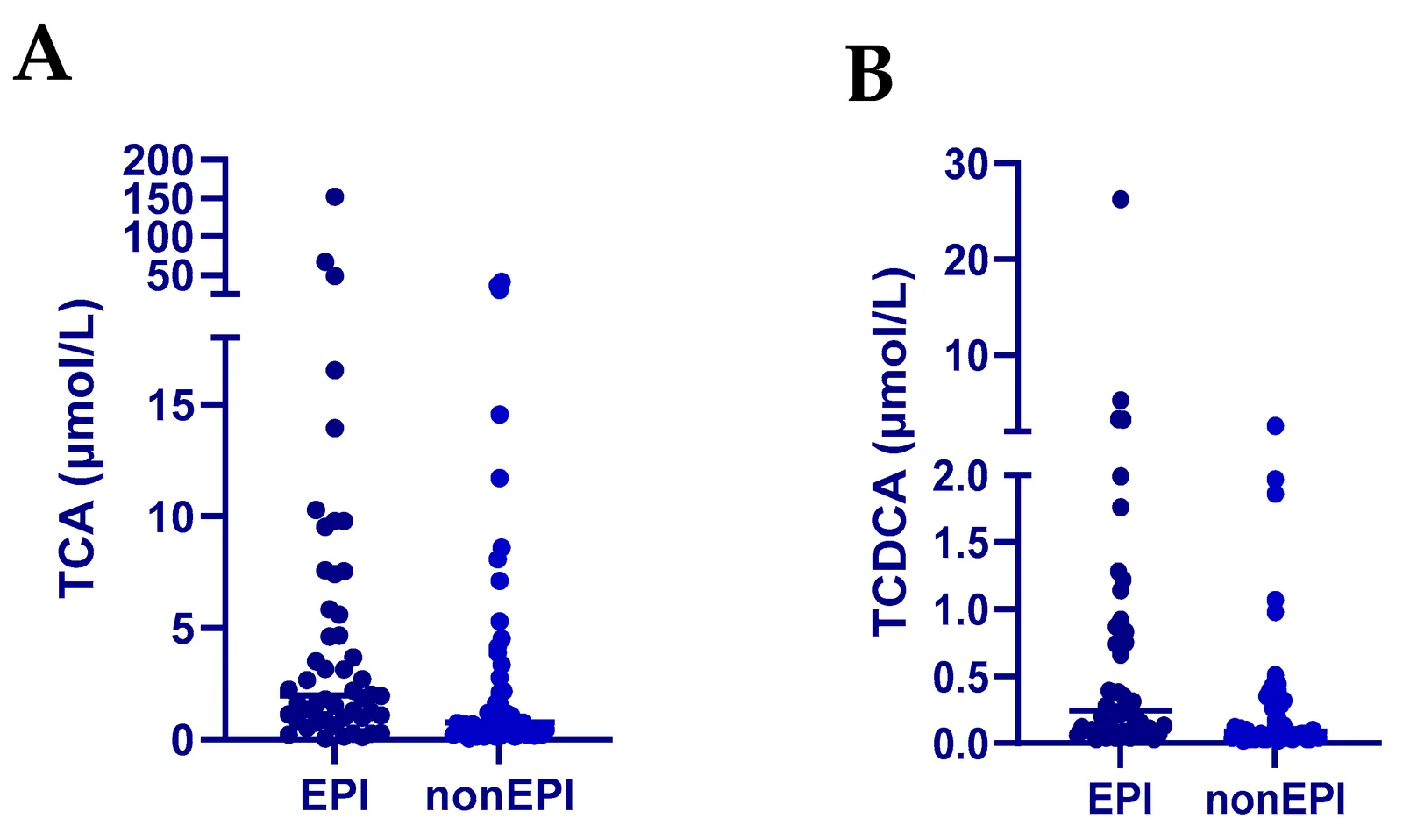
Serum concentrations of taurocholic acid and taurochenodeoxycholic acid: This figure shows serum concentrations of taurocholic acid (**A**) and taurochenodeoxycholic acid (**B**) in 50 dogs with EPI and 49 dogs without EPI of various breeds. Taurocholic acid and taurochenodeoxycholic acid were both significantly higher in dogs with EPI than in those without.

Serum taurocholic acid (TCA) concentrations were significantly different between the two groups of dogs, with dogs with EPI having a median of 2.0 µmol/L (range: 0.1 to 151.9 µmol/L) and dogs without EPI having a median of 0.8 µmol/L (range: 0.1 to 40.9 µmol/L; *p*-value: 0.0120; Table 1, Figure 5). Serum TCA concentrations were statistically significantly different between the four groups of dogs (*p*-value: 0.006). However, Dunn’s multiple comparison analyses only showed a significant difference between non-EPI-GSD and EPI-non-GSDs.

Serum taurochenodeoxycholic acid (TCDCA) concentrations were significantly different between the two groups of dogs, with dogs with EPI having a median of 0.25 µmol/L (range: 0.03 to 26.23 µmol/L) and dogs without EPI having a median of 0.09 µmol/L (range: 0.02 to 2.6 µmol/L; *p*-value: 0.0040; Table 1, Figure 5). Serum TCDCA concentrations were statistically significantly different between the four groups of dogs (*p*-value: 0.014). However, Dunn’s multiple comparison analyses only showed a significant difference between non-EPI-GSD and EPI-non-GSDs.

Serum tauromuricholic acid (TMCA) and taurodeoxycholic acid (TDCA) concentrations were not significantly different between the two groups of dogs. These bile acid concentrations were also not statistically significantly different between the four groups of dogs.

### 3.7. Bile Acids Below Limits of Detection

A variety of the bile acids analyzed showed concentrations below the level of detection for all samples, regardless of group and thus data were not further analyzed. These included tauroursodeoxycholic acid (TUDCA), taurohyodeoxycholic acid (THDCA), taurolithocholic acid-3-sulfate (TLCA-3S), cholic acid-3-sulfate (CA-3S), glycolithocholic acid (GLCA), and hyocholic acid (HCA).

Furthermore, for a variety of bile acids analyzed, most of the serum samples showed concentrations below the detection limit and once again data for these bile acids were not further analyzed: glycolithocholic acid-3-sulfate (GLCA-3S), ursodeoxycholic acid-3-sulfate (UDCA-3S), chenodeoxycholic acid-3-sulfate (CDCA-3S), lithocholic acid-3-sulfate (LCA-3S), tauroursodeoxycholic acid-3-sulfate (TUDCA-3S), glycoursodeoxycholic acid-3-sulfate (GUDCA-3S), glycodeoxycholic acid (GDCA), taurolithocholic acid (TLCA), lithocholic acid (LCA), and hyodeoxycholic acid (HDCA). The number of samples in each group falling below the limit of detection are shown in Supplementary Table S1.

## 4. Discussion

Several of the bile acids that we quantified in the serum samples were below the detection limit of the assay for all of the samples from both groups of dogs, those with EPI and those without EPI. These included taurohyodeoxycholic acid (THDCA), taurolithocholic acid-3-sulfate (TLCA-3S), deoxycholic acid-3-sulfate (DCA-3S), cholic acid-3-sulfate (CA-3S), glycolithocholic acid (GLCA), and hyocholic acid (HCA). For several other BAs, many, but not all, serum samples had results below the detection limit for the assay, including chenodeoxycholic acid-3-sulfate (CDCA-3S), glycolithocholic acid-3-sulfate (GLCA-3S), ursodeoxycholic acid-3-sulfate (UDCA-3S), chenodeoxycholic acid-3-sulfate (CDCA-3S), lithocholic acid-3-sulfate (LCA-3S), tauroursodeoxycholic acid-3-sulfate (TUDCA-3S), glycoursodeoxycholic acid-3-sulfate (GUDCA-3S), glycochenodeoxycholic acid (GCDCA), glycodeoxycholic acid (GDCA), taurolithocholic acid (TLCA), lithocholic acid (LCA), and hyodeoxycholic acid (HDCA).

We were unable to measure any significant concentrations of sulfated bile acids in any of the groups of dogs. Sulfated bile acids are formed in hepatocytes and are important for bile acid detoxification, as the sulfated bile acids are more water soluble than non-sulfated bile acids [10]. Since these sulfated bile acids are directly excreted through the bile into the small intestines from where very little is re-absorbed, measurable amounts of sulfated bile acids in the serum would only be expected in patients with cholestasis, which does not occur in dogs with EPI [10]. Thus, finding unmeasurable concentrations of sulfated bile acids was expected.

Serum total bile acid concentrations were significantly higher in dogs with EPI than in dogs without EPI. Higher total bile acid concentrations have also been reported in the serum of human patients with chronic pancreatitis [9]. While this relationship was not significant for the subgroups of dogs (i.e., the only significant difference shown for subgroups was between non-EPI-GSD and EPI-non-GSD; *p*-value: 0.0096), EPI-GSD had the highest median serum total bile acid concentration (4.7 µmol/L), followed by EPI-non-GSD (3.1 µmol/L), non-EPI-non-GSD (2.5 µmol/L), and non-EPI-GSD (0.8 µmol/L). Thus, the difference between the EPI group and the non-EPI group appeared to be much bigger in GSDs than in dogs of other breeds, but this difference did not reach significance. There are several reasons why serum total bile acids could be higher in dogs with EPI than in non-EPI dogs. Dogs with EPI are predisposed to hepatic lipidosis and even mild hepatic lipidosis reduces the liver’s ability to clear portal bile acids on first pass, resulting in increased serum bile acid concentrations [11]. It should be noted that much higher serum total bile acid concentrations averaging 30 µmol/L (±SD: 33 µmol/L) in healthy adult dogs and measured by similar LC-MS/MS methods were previously reported [12]. However, we are limited in our ability to directly compare between assays because these assays have not previously been cross-validated and may thus give different absolute concentrations. It is worth noting, however, that the concentrations we report here for dogs are similar to those reported for human patients and healthy control subjects using the same method [8,9]. Small intestinal bacterial overgrowth (SIBO) has previously been reported in dogs with EPI and while the term SIBO is no longer being used in dogs, the term has been replaced by intestinal dysbiosis. Intestinal dysbiosis could lead to increased formation of unconjugated bile acids in the intestinal lumen, leading to increased absorption of such unconjugated bile acids and in turn increased serum concentrations of unconjugated bile acids. However, while serum concentrations of two of the unconjugated bile acids, cholic acid (CA) and chenodeoxycholic acid (CDCA), were significantly higher in dogs with EPI than in those without EPI, both CA and CDCA in dogs with EPI were similar to concentrations seen in a previous study of healthy dogs, suggesting that these unconjugated bile acids do not play a major role in dogs with EPI [12]. Furthermore, Karakus et al. found that serum CA was above the detection limit in only eight out of 40 healthy adult dogs and represented approximately 12% of the total measured serum BA pool [12]. They also found that serum CDCA represented a negligible 1.6% of the total serum BA pool with an average concentration of 0.473 µmol/L. In the current study, dogs with EPI had 7.9% of the total serum BA pool represented by CA and 1.4% represented by CDCA, which more closely resembles healthy dogs than our non-EPI group.

In human medicine, it has also been speculated that an increase in serum total bile acid concentrations in individuals with exocrine pancreatic malfunction may be due to trapping bile acids in the small intestine, due to a lack of fat digestion, which in turn leads to an increase in hepatic synthesis of bile acids [13,14].

Similarly to serum total bile acid concentrations, serum primary bile acid concentrations were significantly higher in dogs with EPI than in non-EPI dogs, while serum secondary bile acid concentrations were not different between the two groups. There was also a significant difference in primary bile acid concentrations when evaluating the four subgroups, but post hoc analysis was only able to identify a significant difference between non-EPI-GSD and EPI-non-GSD (*p*-value: 0.0069), suggesting that the size of the subgroups was not large enough to identify a significant difference. Accordingly, median serum primary bile acid concentrations were highest in EPI-GSD (3.7 µmol/L), followed by 3.1 µmol/L for EPI-non-GSD, 2.0 µmol/L for non-EPI-non-GSD, and 0.7 µmol/L for non-EPI-GSD.

As secondary bile acids are a reflection of bacterial metabolism in the intestinal lumen through dehydroxylation, deconjugation, and epimerization, there are several possible explanations for not being able to identify a statistically significant difference between EPI and non-EPI dogs. Firstly, this could suggest that in dogs with EPI, bacterial species that form secondary bile acids are either unaltered or that bacterial formation of secondary bile acids occurs mainly in the colon, after reabsorption of the bile acid pool from the intestinal lumen. Another possible explanation is that dogs that did not have EPI in this study did in fact also have intestinal dysbiosis. All samples had been submitted to the GI Lab at Texas A&M University, likely because the dogs showed clinical signs of gastrointestinal disease. Thus, it is possible that non-EPI dogs had intestinal dysbiosis leading to similar alterations of the intestinal microbiome in both EPI and non-EPI dogs. However, the dogs selected for the non-EPI group had serum cobalamin and folate concentrations within the reference interval, suggesting that they did not have significant small intestinal disease, which one would expect in dogs with intestinal dysbiosis. Since primary bile acids were significantly higher in dogs with EPI than in non-EPI dogs and secondary bile acid concentrations were not significantly different, these results were mirrored by the ratio of primary to secondary bile acids, but there is no additional information that can be gleaned from this ratio when compared to the difference in primary bile acids between the groups. It is worth noting that a significant increase in primary to secondary BA ratio has also been observed in human patients with EPI that is due to severe chronic pancreatitis [9]. In that study, BA concentrations were measured not only in plasma but also simultaneously in small intestinal contents, revealing an altered BA secretion in EPI. Most of the patients in that study had a history of bile duct stenosis. Therefore, it was hypothesized that primary BA that had been synthesized in the liver were re-absorbed into the bile duct before reaching the small intestine through the so-called cholangiohepatic shunt of BA secretion [15]. However, this explanation appears unlikely for the dogs in this study. Almost all human patients with EPI develop EPI as a sequela to chronic pancreatitis and thus frequently have biliary stenosis. In sharp contrast, GSDs with EPI do not show any significant inflammation in the pancreas or surrounding tissues. If in fact biliary stenosis secondary to biliary inflammation were to have caused the bile acid dysmetabolism observed here, those changes should only have been apparent in dogs with EPI in non-GSD breeds. Instead, the changes we observed were more pronounced in the EPI-GSDs than in the EPI-non-GSDs.

Dogs have two primary bile acids, cholic acid and chenodeoxycholic acid, and the total concentration of both were significantly higher in dogs with EPI than in dogs without EPI. However, overall total serum chenodeoxycholic acid concentrations were much lower than those of total serum cholic acid concentrations (median serum total cholic acid concentrations in dogs with EPI 2.8 µmol/L; median serum total chenodeoxycholic acid concentrations in dogs with EPI 0.33 µmol/L). Bile acids containing a 12α-hydroxyl group, such as cholic acid, are more predominant in dogs than bile acids without a 12α-hydroxyl group, such as chenodeoxycholic acid, because the liver favorably synthesizes and secretes 12α-hydroxylated bile acids through sterol 12-alpha-hydroxylase (CYP8B1) activity in de novo bile acid synthesis [16,17,18].

Muricholic acid is a primary bile acid in rodents and is not usually synthesized in quantifiable amounts in canine hepatocytes. While small amounts of muricholic acid were identified in the serum of some dogs and serum muricholic acid concentrations were significantly higher in dogs with EPI than in dogs without EPI, both had very low median concentrations precluding a more detailed assessment. Isolated dogs had, however, extremely high amounts of serum muricholic acid concentrations (e.g., 9.55 µmol/L), which would suggest significant bile acid dysmetabolism in these isolated dogs. It is unclear whether this could stem from differences in luminal microbial biotransformation reactions (including 6-position hydroxylation, oxidation, and epimerization); an individual difference in bile acid synthesis in hepatocytes or, less likely, could be related to ingestion of a rodent or feed containing rodent tissues or feces and hepatic recirculation of the bile acid pool from that rodent. Other studies have also found low amounts of α-, β-, and ω-MCA in a limited number of serum samples from healthy adult dogs and in fecal samples from dogs [12,17,19]. Also, dogs with chronic inflammatory enteropathy have been reported to have significantly higher fecal concentrations of β-MCA, reaching as high as 41.83 µg/g feces in one study [17].

When assessing the specific bile acids, many of them showed concentrations below the detection limit of the assay. Those that showed measurable concentrations in most dogs included taurochenodeoxycholic acid, taurocholic acid, ursodeoxycholic acid, and cholic acid, all of which were significantly higher in dogs with EPI. The relatively high serum concentrations of taurocholic acid and taurocheonodeoxycholic acid are not surprising as taurine conjugation is the primary type of bile acid conjugation in dogs and the majority of bile acids excreted in bile are re-absorbed in the distal small intestine.

The median serum ursodeoxycholic acid concentration was 0.00 µmol/L in both dogs with EPI and those without, suggesting low concentrations in both groups of dogs, despite a statistically significant difference. Another study found increased fecal UDCA concentrations in dogs with treated EPI compared to healthy control dogs [20]. The same study also found significantly lower 7α-dehydroxylated bile acids, DCA and LCA, in the feces of treated EPI dogs compared to healthy controls. This suggests that dogs with EPI may have lower 7α-dehydroxylating bacterial enzyme activity in the lumen, leading to increased CDCA substrate availability for hydroxysteroid dehydrogenase bacterial enzymes to form UDCA. Indeed, all untreated dogs with EPI and a subgroup of dogs with treated EPI had fecal dysbiosis characterized by loss of one of the key bacterial species with 7α-dehydroxylating activity, *Peptacetobacter* (*Clostridium*) *hiranonis* [20].

Thus, it appears that the amounts of both primary bile acids, cholic acid and chenodeoxycholic acid, are significantly increased in the serum of dogs with EPI, which is associated with a significant increase in total primary bile acids and, in turn, total bile acids. There are several possible explanations for this finding that were, however, not further explored in the current study but represent areas of future studies in this field. The most simplistic explanation would be an upregulation of primary bile acid synthesis in hepatic cells. Both primary bile acids are synthesized from cholesterol in hepatocytes and the rate-limiting enzyme for the synthesis of both is cholesterol 7 alpha-hydroxylase (CYP7A1) [21]. The activity of CYP7A1 is primarily modulated by the concentration of primary bile acids in the intestines as bile acids bind to the farnesoid X receptor (FXR) [22]. Thus, increased amounts of bile acids in the intestinal lumen should be self-regulating. It is interesting to speculate that undigested chyme could counteract this decrease in bile acid synthesis mediated through FXR, for example by trapping bile acids. While an impact of undigested chyme in the intestinal lumen has not previously been demonstrated to have an impact on CYP7A1 activity, diet has been demonstrated to have such an effect [23]. Similarly, several of the cholic acid species that were found to be increased in dogs with EPI in the current study act as FXR antagonists, leading to a decrease in fibroblast growth factor 15 and fibroblast growth factor 19 (FGF15/19), which in turn decreases the inhibition of bile acid synthesis that is usually achieved through FGF15/19 [24]. Thus, these FXR antagonists would directly stimulate bile acid synthesis in hepatocytes [24]. Finally, but less likely, the reabsorption of bile acids from the intestinal lumen and thus hepatic recirculation of bile acids may be more efficient in dogs with EPI. This could also be related to the dysbiosis of the intestinal microbiome, as has previously been described in dogs with EPI and may explain increases seen in the bile acids UDCA and MCA.

Overall, German shepherd dogs did not show strong differences in serum bile acids compared to other breeds of dogs in either EPI or non-EPI dogs. This suggests that the underlying etiology of pancreatic dysfunction, PAA or chronic pancreatitis, does not significantly impact the changes seen in bile acid metabolism seen in dogs with EPI, and that GSDs without EPI are not inherently predisposed to significant bile acid dysmetabolism.

There were several limitations to this study, including the retrospective method of sample collection. The non-EPI group consisted of clinical patients with suspected GI disease rather than healthy controls, introducing confounding variables related to ileal malabsorption or subclinical liver disease in the comparison group. Patient history was not available for any of the dogs included in the study, and therefore result interpretation could be limited by the unintentional inclusion of dogs with comorbidities or medications that would affect serum bile acid concentrations. This also limited our ability to tease out contributions of dysbiosis or gastrointestinal disease to the differences seen between groups. However, the intention of including only dogs with serum cobalamin and folate concentrations within their respective reference intervals for the non-EPI group was to exclude those with overt GI disease. This method of sampling may have decreased sampling bias and helped represent the true target population.

One of the important questions our study raises is what the clinical impact of the bile acid dysmetabolism could be in dogs with EPI. Dogs with hepatobiliary disease leading to hepatic failure often have severely increased serum total bile acid concentrations, which often reach 5–10-fold the upper limit of the reference interval [25]. However, in these dogs the increased serum total bile acid concentrations are not related to any specific clinical signs or complications. Thus, it would be unlikely that the increases in dogs with EPI observed here would be associated with such clinical signs or complications.

## 5. Conclusions

Serum concentrations of cholic acid and chenodeoxycholic acid are increased in dogs with EPI, regardless of the breed of dog affected and thus likely regardless of the underlying cause of EPI. It is important to note that our inability to detect a statistically significant difference between the subgroups of dogs may have been due to a limited sample size. The changes in serum concentrations of cholic acid and chenodeoxycholic acid are further associated with a significant increase in serum concentrations of total primary bile acids and total bile acids. The physiological and clinical consequences of this bile acid dysmetabolism in dogs with EPI will need to be further explored.

## Figures and Tables

**Table 1:** This table shows the comparison of serum bile acid concentrations between EPI dogs and non-EPI dogs that were statistically significantly different. In addition, results for serum total secondary bile acids are shown. All shown datasets failed normality testing and median and ranges are shown for each.

Bile Acid Analyzed	EPI µmol/L	non-EPI µmol/L	*p*-Value
Total BA	4.1 (0.2–196.0)	1.6 (0.1–47.7)	0.0022
Primary BA	3.7 (0.2–40.5)	0.7 (0.2–38.4)	0.0161
Secondary BA	0.04 (0.00–3.76)	0.04 (0.00–13.92)	0.9399
P/S BA ratio	73 (3–4148)	11 (2–1213)	0.0152
T-conjugated BA	2.5 (0.1–182.2)	1.1 (0.1–45.9)	0.0205
TCDCA	0.25 (0.03–26.23)	0.09 (0.02–2.6)	0.0040
TCA	2.0 (0.1–151.9)	0.8 (0.1–40.9)	0.0120
UDCA	0.00 (0.00–2.27)	0.00 (0.00–0.15)	0.0023
CDCA	0.06 (0.00–7.44)	0.00 (0.00–0.15)	<0.0001
CA	0.33 (0.00–55.34)	0.06 (0.00–3.38)	<0.0001
MCA	0.03 (0.00–9.55)	0.00 (0.00–0.41)	0.0018

## Data Availability

The original data presented in the study are openly available in Zenodo at https://doi.org/10.5281/zenodo.22052402.

## Supplementary Materials

The following supporting information can be downloaded at https://www.mdpi.com/article/10.3390/ani16182941/s1, Table S1: Number of dogs for which the specific bile acid listed fell below the limit of detection.

## Abbreviations

The following abbreviations are used in this manuscript:

BA	bile acids
cTLI	canine trypsin-like immunoreactivity
CA	cholic acid
CA-3S	cholic acid-3-sulfate
CDCA	chenodeoxycholic acid
GCDCA	glycochenodeoxycholic acid
GDCA	glycodeoxycholic acid
CDCA-3S	chenodeoxycholic acid-3-sulfate
CYP7A1	cholesterol 7 alpha-hydroxylase
CYP8B1	sterol 12-alpha-hydroxylase
DCA	deoxycholic acid
EPI	exocrine pancreatic insufficiency
FGF15	fibroblast growth factor 15
FGF19	fibroblast growth factor 19
GCA	glycocholic acid
GI-Lab	Gastrointestinal Laboratory
GLCA	glycolithocholic acid
GLCA-3S	glycolithocholic acid-3-sulfate
GSD	German shepherd dog
GUDCA	glycoursodeoxycholic acid
GUDCA-3S	glycoursodeoxycholic acid-3-sulfate
HCA	hyocholic acid
HDCA	hyodeoxycholic acid
LCA	lithocholic acid
LCA-3S	lithocholic acid-3-sulfate
MCA	muricholic acid
Non-EPI	non-EPI
Non-GSD	non-German-shepherd dog
PAA	pancreatic acinar atrophy
PERT	pancreatic enzyme replacement therapy
TCA	taurocholic acid
TCDCA	taurochenodeoxycholic acid
TDCA	taurodeoxycholic acid
THDCA	taurohyodeoxycholic acid
TLCA	taurolithocholic acid
TLCA-3S	taurolithocholic acid-3-sulfate
TMCA	tauromuricholic acid
TUDCA	tauroursodeoxycholic acid
TUDCA-3S	taurodeoxycholic acid-3-sulfate
UDCA	ursodeoxycholic acid
UDCA-3S	ursodeoxycholic acid-3-sulfate

## References

[1] Cridge H., Williams D.A., Barko P.C. (2024). Exocrine pancreatic insufficiency in dogs and cats. J. Am. Vet. Med. Assoc..

[2] Westermarck E., Batt R.M., Vaillant C., Wiberg M. (1993). Sequential study of pancreatic structure and function during development of pancreatic acinar atrophy in a German shepherd dog. Am. J. Vet. Res..

[3] Westermarck E., Saari S., Wiberg M. (2010). Heritability of exocrine pancreatic insufficiency in German shepherd dogs. J. Vet. Intern. Med..

[4] Westermarck E., Wiberg M. (2012). Exocrine pancreatic insufficiency in the dog: Historical background, diagnosis, and treatment. Top. Companion Anim. Med..

[5] Westermarck E., Siltanen R., Maijala R. (1993). Small intestinal bacterial overgrowth in seven dogs with gastrointestinal signs. Acta Vet. Scand..

[6] Barko P.C., Rubin S.I., Swanson K.S., McMichael M.A., Ridgway M.D., Williams D.A. (2023). Untargeted analysis of serum metabolomes in dogs with exocrine pancreatic insufficiency. Animals.

[7] Melgarejo T., Williams D.A., O’Connell N.C., Setchell K.D. (2000). Serum unconjugated bile acids as a test for intestinal bacterial overgrowth in dogs. Dig. Dis. Sci..

[8] Humbert L., Maubert M.A., Wolf C., Duboc H., Mahé M., Farabos D., Seksik P., Mallet J.M., Trugnan G., Masliah J. (2012). Bile acid profiling in human biological samples: Comparison of extraction procedures and application to normal and cholestatic patients. J. Chromatogr. B.

[9] Humbert L., Rainteau D., Tuvignon N., Wolf C., Seksik P., Laugier R., Carrière F. (2018). Postprandial bile acid levels in intestine and plasma reveal altered biliary circulation in chronic pancreatitis patients. J. Lipid Res..

[10] Alnouti Y. (2009). Bile Acid sulfation: A pathway of bile acid elimination and detoxification. Toxicol. Sci..

[11] Adamama-Moraitou K.K., Rallis T.S., Papazoglou L.G., Papasteriadis A., Roubies N., Kaldrimidou H., Leontides L.S. (2004). Liver biochemical and histopathological findings in dogs with experimentally induced exocrine pancreatic insufficiency. Can. J. Vet. Res..

[12] Karakus E., Proksch A.-L., Moritz A., Geyer J. (2024). Quantitative bile acid profiling in healthy adult dogs and pups from serum, plasma, urine, and feces using LC-MS/MS. Front. Vet. Sci..

[13] Goodchild M.C., Murphy G.M., Howell A.M., Nutter S.A., Anderson C.M. (1975). Aspects of bile acid metabolism in cystic fibrosis. Arch. Dis. Child..

[14] Robb T.A., Davidson G.P., Kirubakaran C. (1985). Conjugated bile acids in serum and secretions in response to cholecystokinin/secretin stimulation in children with cystic fibrosis. Gut.

[15] Hofmann A.F. (1989). Current concepts of biliary secretion. Dig. Dis. Sci..

[16] Kakimoto T., Kanemoto H., Fukushima K., Ohno K., Tsujimoto H. (2017). Effect of a high-fat-high-cholesterol diet on gallbladder bile acid composition and gallbladder motility in dogs. Am. J. Vet. Res..

[17] Comito R., Porru E., Interino N., Conti M., Terragni R., Gotti R., Candela M., Simoni P., Roda A., Fiori J. (2023). Metabolic Bile acid profile impairments in dogs affected by chronic inflammatory enteropathy. Metabolites.

[18] Németh K., Sterczer Á., Kiss D.S., Lányi R.K., Hemző V., Vámos K., Bartha T., Buzás A., Lányi K. (2024). Determination of bile acids in canine biological samples: Diagnostic significance. Metabolites.

[19] Lin Q., Tan X., Wang W., Zeng W., Gui L., Su M., Liu C., Jia W., Xu L., Lan K. (2020). Species differences of bile acid redox metabolism: Tertiary oxidation of deoxycholate is conserved in preclinical animals. Drug Metab. Dispos..

[20] Blake A.B., Guard B.C., Honneffer J.B., Lidbury J.A., Steiner J.M., Suchodolski J.S. (2019). Altered microbiota, fecal lactate, and fecal bile acids in dogs with gastrointestinal disease. PLoS ONE.

[21] Chiang J.Y., Ferrell J.M. (2020). Up to date on cholesterol 7 alpha-hydroxylase (CYP7A1) in bile acid synthesis. Liver Res..

[22] Lee E.A., Lee D.I., Kim H.Y., Ahn S.-H., Seong H.-R., Jung W.H., Kim K.Y., Kim S., Rhee S.D. (2018). Cyp7a1 is continuously increased with disrupted Fxr-mediated feedback inhibition in hypercholesterolemic TALLYHO/Jng mice. Biochim. Biophys. Acta Mol. Cell Biol. Lipids.

[23] Moon M.S., Lee M.S., Kim C.T., Kim Y. (2007). Dietary chitosan enhances hepatic CYP7A1 activity and reduces plasma and liver cholesterol concentrations in diet-induced hypercholesterolemia in rats. Nutr. Res. Pract..

[24] Sun L., Xie C., Wang G., Wu Y., Wu Q., Wang X., Liu J., Deng Y., Xia J., Chen B. (2018). Gut microbiota and intestinal FXR mediate the clinical benefits of metformin. Nat. Med..

[25] Center S.A., Baldwin B.H., Erb H.N., Tennant B.C. (1985). Bile acid concentrations in the diagnosis of hepatobiliary disease in the dog. J. Am. Vet. Med. Assoc..

