# Supplementary material for: Serum Bile Acid Concentrations in Dogs with Exocrine Pancreatic Insufficiency
Source: Animals (Basel). 2026 Sep 18;16(18):2941. doi: 10.3390/ani16182941 (PMC13603600; doi:10.3390/ani16182941)
Supplement: Supplementary file 1 [file animals-16-02941-s001.zip › animals-4543548-supplementary.pdf]

Supplementary table

	EPI	nonEPI
glycolithocholic acid-3-sulfate (GLCA-3S)	49/50	48/49
ursodeoxycholic acid-3-sulfate (UDCA-3S)	41/50	44/49
chenodeoxycholic acid-3-sulfate (CDCA-3S)	50/50	48/49
lithocholic acid-3-sulfate (LCA-3S)	50/50	48/49
tauroursodeoxycholic acid-3-sulfate (TUDCA-3S)	46/50	46/49
glycoursodeoxycholic acid-3-sulfate (GUDCA-3S)	49/50	47/49
glycodeoxycholic acid (GDCA)	44/50	44/49
taurolithocholic acid (TLCA)	45/50	46/49
lithocholic acid (LCA)	49/50	48/49
hyodeoxycholic acid (HDCA)	50/50	47/49

Table S1. Number of dogs for which the specific bile acid listed fell below the limit of detection.
